# *Monascus* red pigments alleviate high‐fat and high‐sugar diet‐induced NAFLD in mice by modulating the gut microbiota and metabolites

**DOI:** 10.1002/fsn3.4208

**Published:** 2024-05-21

**Authors:** Wenyan Gao, Xinghao Chen, Shaokang Wu, Lu Jin, Xu Chen, Genxiang Mao, Xiaoqing Wan, Wenmin Xing

**Affiliations:** ^1^ School of Pharmacy Hangzhou Medical College Hangzhou China; ^2^ Department of Pharmacy Qingdao Sixth People's Hospital Qingdao China; ^3^ Zhejiang Provincial Key Lab of Geriatrics Zhejiang Hospital Hangzhou China

**Keywords:** gut microbiota, metabolites, *Monascus* red pigments, NAFLD

## Abstract

*Monascus* red pigments (MRP) may have benefits against NAFLD with an unclear mechanism. This study aimed to explore the protective effect of MRP supplementation against NAFLD through regulation of gut microbiota and metabolites. The C57BL/6 mice animals were randomly allocated into the normal diet (NC), HFHS diet‐induced NAFLD model, and MRP intervention group fed with HFHS diet. Serum lipid profiles and liver function parameters were measured. Liver and colon histopathology analysis was conducted to determine the injury in the liver and colon. 16S rRNA gene sequencing was employed to analyze gut microbial composition from fecal samples. Untargeted metabonomics was performed to analyze changes in metabolites in serum and fecal samples. MRP supplementation significantly improved the HFHS‐induced alterations in body weight, lipid profiles, and liver function (*p* < .01). MRP supplementation decreased the abundance of *Akkermansia*, *Candidatus saccharimonas*, *Dubosiella*, and *Oscillibacter*, while increasing *Lactobacillus*, *Lachnospiraceae NK4A136 group*, and *Rikenella* in mice fed the HFHS diet. Furthermore, MRP supplementation improved the serum and fecal metabolic profiles induced by the HFHS diet, primarily involving the arachidonic acid metabolism, unsaturated fatty acid biosynthesis, and adipocyte lipolysis pathways. Liver function and lipid profiles were closely associated with the abundance of *Lactobacillus*, *Streptococcus*, *Oscillibacter*, *Akkemansia*, and *Desulfovibrio* (*p* < .01). These findings revealed that MRP supplementation may help restore gut microbiota composition and balance its metabolites, thereby improving NAFLD. This study presents a novel outlook on the potential benefits of MRP supplementation in ameliorating NAFLD and supports the application of MRP as a new functional food.

## Graphical abstract

MRP supplementation could improve HFHS‐induced liver enzymes, lipid profiles, and inflammation factors. Moreover, MRP supplementation restored HFHS‐caused disorders of the gut microbiota and regulated the serum and fecal metabolic profiles, especially the arachidonic acid metabolism, biosynthesis of unsaturated fatty acids, and lipolysis in the adipocytes pathway, to ameliorate the NAFLD induced by the HFHS diet.

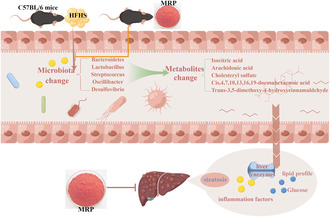

## 1 INTRODUCTION

Nonalcoholic fatty liver disease (NAFLD) is characterized by the accumulation of lipids in the liver, which can progress to hepatic steatosis (HS), steatohepatitis, cirrhosis, and even hepatocellular carcinoma (HCC) in association with NAFLD (Craven et al., 2020; Nawrot et al., 2021; Ponziani et al., 2019). The incidence of NAFLD has been alarmingly rising due to changes in dietary patterns, affecting approximately 20%–30% of adults in North America, 80% of individuals with obesity, and nearly all individuals diagnosed with type 2 diabetes (T2D) (Aron‐Wisnewsky et al., 2020; Bauer et al., 2022). The gut microbiota consists of a diverse range of microorganisms, including bacteria, fungi, viruses, archaea, and protists (Turnbaugh et al., 2007), closely related to host health (Afzaal et al., 2022). The liver maintains bile secretion into the intestine, while bile is first released into the biliary system and then directly into the small intestine. Thus, maintaining liver homeostasis benefits from the presence of commensal gut microbes, while an imbalance in the gut microbiota can potentially lead to liver damage (Lang & Schnabl, 2020). Furthermore, metabolites produced by the microbiota, such as short‐chain fatty acids (SCFAs), could elevate inflammatory factors and increase intestinal epithelial barrier permeability, thereby exacerbating the progression of NAFLD.

The utilization of *Monascus* spp. in the generation of fermented red yeast rice has been a long‐standing practice in China with significant applications in both the food and pharmaceutical industries (Huang et al., 2023). Studies have indicated that *Monascus*‐fermented extracts can help alleviate elevated levels of serum triglycerides (TG) and cholesterol (TC) in rats that are fed a high‐fat diet (Li et al., 2023; Pyo & Seong, 2009). *Monascus* pigments, encompassing yellow, red, and orange pigments, are widely used in the food and health supplements industry in China, Southeast Asian areas, Japan, and Indonesia (Chen et al., 2017; Xu Xiong et al., 2015). These pigments have been found to have various biological effects, including potential anti‐atherosclerotic properties and the ability to decrease body fat content. For example, the orange pigment has been shown to possess anti‐atherosclerotic properties, making it a potential natural alternative for use in clinical trials (Kim & Ku, 2018). Moreover, the *Anka monascus* pigment has been linked to a reduction in blood lipid levels. *Monascus* red pigment (MRP) has been associated with various beneficial biological and therapeutic effects, including anticancer, antioxidant, and anti‐obesity effects (Kim et al., 2007; Kurokawa et al., 2021). Nevertheless, the specific lipid‐ and fat‐reducing characteristics of MRP have not been well evaluated, and the underlying mechanisms remain largely unexplored. Therefore, further research is warranted to gain a comprehensive insight into the potential benefits and mechanisms related to MRP in the reduction of lipid and fat.

In the present study, we investigate the potential benefits of MRP supplementation in alleviating NAFLD induced by a high‐fat, high‐sugar (HFHS) diet in mice, as well as its impact on gut microbiota and metabolites. C57BL/6 mice were employed as an animal model to assess the improvement effect of MRP supplementation on NAFLD. Changes in body weight, serum lipid profiles, blood glucose, systematic inflammatory factors, liver function parameters, and colon and liver pathology were observed in each sample. Furthermore, changes in the composition of the gut microbiota and the profile of metabolites in fecal and serum samples were explored using 16S DNA sequencing and untargeted metabonomics detection. The correlation between changes in gut microbiota, metabolites, liver function parameters, and lipid profiles was also investigated. These findings provide an experimental foundation for the potential utilization of MRP in the treatment and prevention of NAFLD. Additionally, our results highlighted the promising therapeutic potential of MRP in NAFLD and other disorders associated with intestinal microbial dysbiosis.

## 2 MATERIALS AND METHODS

### 2.1 Animals

Male C57BL/6J mice weighing 20 ± 2 g were obtained from Hangzhou Medical College Animal Technology Co., Ltd. The mice were housed in controlled conditions with a temperature of 22 ± 1°C, a relative humidity of 60 ± 10%, and a 12‐h light/dark cycle to standardize their living conditions during the study. The mice were divided into two groups and fed either a normal diet or a HFHS diet purchased from Medicience Co., Ltd. All animal‐related experiments were performed following established protocols and approved guidelines by the Animal Experimental Ethics Committee of Hangzhou Medical College. The mice were randomly divided into three groups, including the control group (NC), the NAFLD model group, and the MRP group. MRPs (lot number: Q22F12N139672) were purchased from Shanghai Yuanye Biotechnology Co., Ltd. (Shanghai, China). Based on a previous study (Zhou et al., 2019), the dose of MRP administered to the mice was set at 40 mg/kg. Except for the control group, NAFLD model mice were fed a HFHS diet for 8 weeks. Blood samples were collected to confirm the development of the NAFLD model. Subsequently, the MRP groups received daily gavage of MRP for 16 weeks, while the NAFLD model group continued to be fed the HFHS diet and the NC group remained a standard diet. At the end of the 16‐week intervention, the weight of each mouse was assessed and anesthetized with a 1% solution of pentobarbital sodium. Blood, fecal, liver, and intestinal tissues were rapidly collected.

### 2.2 Assessment of serum lipid and liver function index profiles

We measured serum lipid profiles, including TG, TC, low‐density lipoproteincholesterol (LDL‐C), and high‐density lipoproteincholesterol (HDL‐C), as well as markers of inflammation and glucose tolerance. Furthermore, liver function parameters, such as alanine aminotransferase (ALT) and aspartate aminotransferase (AST), were also evaluated.

### 2.3 Histopathology

#### 2.3.1 Hematoxylin and eosin (H&E) staining

Liver injury, fibrosis, and colon structure were examined using H&E staining. Fresh liver samples were fixed in 4% paraformaldehyde for 48 h, then embedded in paraffin wax and sectioned into 4 μm sections using a Leica microtome (LEICA RM2016) according to standard histological procedures. The sections were then deparaffinized, stained with hematoxylin–eosin dye, and examined for histopathological changes in the liver under a light microscope (LEICA DM 1000).

#### 2.3.2 Oil red O staining

Liver lipid droplets were observed using oil red O and hematoxylin staining. Liver samples were embedded in an optimal cutting temperature (OCT) compound and stored at −20°C. And then, the samples were sectioned into 8 μm slices using a cryostat (LEICA CM1850) manufactured by Leica. The staining process involved washing the sections three times with pH 7.2 PBS for 5 min each, followed by immersion in 60% isopropanol (2 min). Subsequently, the samples were in oil red O with 60% isopropanol (37°C, 5 min). The sections were washed again in 60% isopropanol for 3 min and then in ddH_2_O before being subjected to hematoxylin staining for 2 min and hydrochloric acid alcohol treatment for 3 s. And then, the sections were sealed with coverslips to observe the pathological change under a microscope (LEICA DM 1000).

### 2.4 16S rDNA gene sequence analysis

Fecal samples were collected from each mice for microbial analysis (*n* = 6). DNA was isolated from fecal samples using the Magnetic Soil and Stool DNA Kit (TIANGEN). The quality and quantity of the extracted DNA were evaluated using 1% agarose gel electrophoresis. To achieve a consistent concentration of 1 ng/μL, the extracted DNA was diluted with sterile water. The 16S rRNA genes were amplified using specific primers with barcode and the sequencing libraries were prepared following the manufacturer's instructions. Unique index codes were assigned to each library for multiplexed sequencing. Prior to sequencing, the library quality was assessed using the Qubit@ 2.0 Fluorometer (Thermo Scientific) and the Agilent Bioanalyzer 2100 system. The Illumina NovaSeq6000 platform was used for sequencing. The UPARSE software package, including the UPARSE‐OTU and UPARSE‐OTUref algorithms, was used for sequence analysis. A representative sequence and taxonomic information were selected and annotated using the RDP classifier (Silva 132 for 16S, UNITE for ITS). For α‐diversity analysis, four diversity metrics were calculated: species abundance (Chao1 and Observed_species indexes) and diversity (Shannon and Simpson indexes). Moreover, β‐diversity was visualized using principal component analysis (PCA) and non‐metric multi‐dimensional scaling in NMDS. The STAMP software was used to confirm differences in the abundance of individual taxa between the two groups. Additionally, quantitative analysis of biomarkers within different groups was performed using the LEfSe method.

### 2.5 Assessment of the fecal and serum metabolite profiles

Fecal and serum samples were collected from each mice and used for untargeted metabolomics analysis (n = 6). A cold extraction solvent (methanol:acetonitrile:water = 2:2:1) was added to 80–100 mg of the sample to extract metabolites from fecal samples. For serum samples, we added 400 μL of cold extraction solvent (methanol/acetonitrile/H_2_O, 2:2:1) to 100–150 μL of sample. The samples were mixed well and incubated on ice for 20 min. The supernatant was collected (14,000 g, 20 min, 4°C), and then the supernatant was passed through a 96‐well protein precipitation plate. The resulting eluate was then dried using a vacuum centrifuge. To analyze the extracts, hydrophilic interaction chromatography coupled with electrospray ionization and quadrupole time‐of‐flight mass spectrometry (Sciex TripleTOF 6600) was employed. The raw MS data underwent conversion to MzXML files using Proteo Wizard MSConvert before being imported into the XCMS software for further analysis.

The PCA and orthogonal partial least squares discriminant analysis (OPLS‐DA) were performed using the SIMCA‐P (version 14.1, Umetrics, Umea, Sweden). To assess the role of each variable in classification, the OPLS‐DA model was used to compute the variable importance in projection (VIP) values. Significance was identified by applying an unpaired Student's *t*‐test. Metabolites were mapped to KEGG pathways by the online Kyoto Encyclopedia of Genes and Genomes (KEGG) database.

### 2.6 Pearson correlation coefficient model

Pearson correlation coefficients were analyzed to assess the relationship between microbial abundance, liver function, lipid profiles, and metabolite integration. The genus‐level microbial abundance was ranked in descending order, and metabolites that showed differential expression between the NC, NAFLD model, and MRP groups were selected. The top 10 microbial genera and the differently expressed metabolites were analyzed using pheatmap packages in the R software. The Pearson correlation coefficient between the abundance of microbiota and the levels of ALT, AST, lipid profiles, and differently expressed metabolites was conducted in R (version 3.5.1).

### 2.7 Statistical analysis

The data were presented as mean ± SD. The continuous variable was calculated by t‐test (PRISM 9.0). Significance was considered as a *p* < .05.

## 3 RESULTS

### 3.1 Improvement of MRP on HFHS‐induced lipid profiles and liver injury in C57BL/6 mice

**FIGURE 1:**
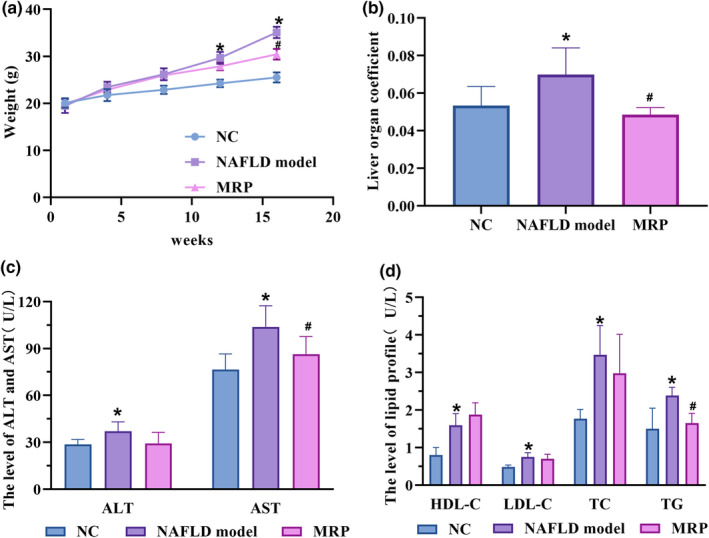
Effects of MRP supplementation on the body weight, liver organ coefficient, serum AST, ALT, and lipid profiles in C57/BL mice fed on a HFHS diet. *p* < .05 was considered to be significant. *, NAFLD model group vs. NC group; #, MRP group vs. NAFLD model group. ALT, alanine aminotransferase; AST, aspartate aminotransferase; HDL‐C, high‐density lipoproteincholesterol; LDL‐C, low‐density lipoprotein‐cholesterol; NC, normal group; TC, total cholesterol; TG, triglycerides.

To assess the impact of MRP supplementation on HFHS‐induced NAFLD, we observed the body weight of mice in each group. The mice were either fed a normal diet or a HFHS diet for 8 weeks, while simultaneously receiving intragastric administration with or without MRP (40 mg/kg) for 16 weeks. We observed that MRP supplementation significantly reduced the mice's body weight in the HFHS group, particularly at 12 and 16 weeks (Figure 1a). Additionally, the liver organ coefficient was notably decreased by MRP supplementation at 16 weeks (Figure 1b). MRP supplementation also resulted in a decrease in elevated ALT and AST induced by the HFHS (Figure 1c), indicating an ameliorative effect on impaired liver function of MRP. Moreover, mice receiving MRP supplementation presented a reversal in the levels of TG, TC, and LDL‐C, which were increased by the HFHS, particularly in the case of TG (*p* < .05) (Figure 1d).

### 3.2 Improvement of MRP on HFHS‐induced hepatic histopathology change in C57BL/6 mice

**FIGURE 2:**
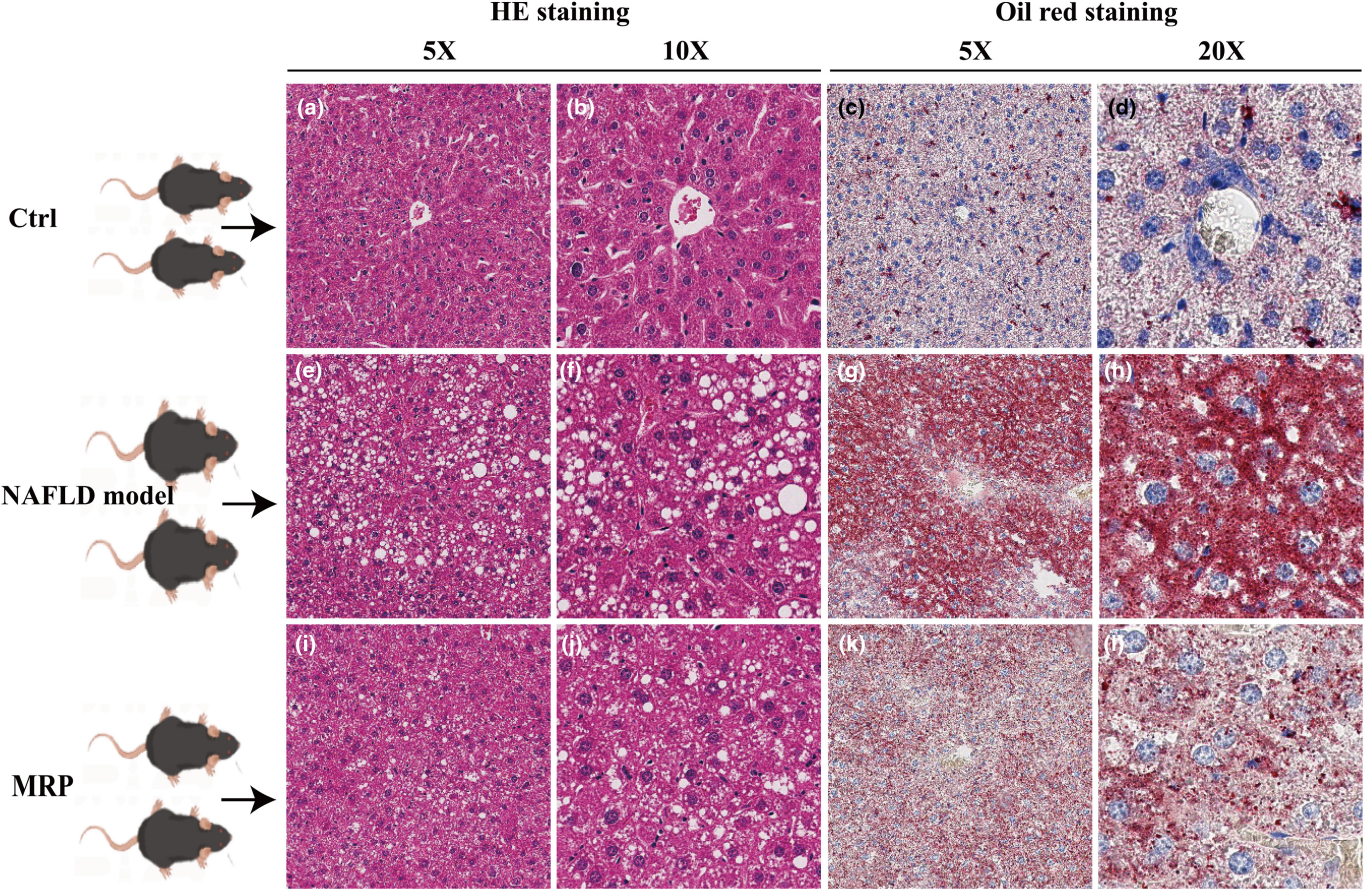
Effects of MRP supplementation on hepatic lipid accumulation in mice fed on a HFHS diet. Histopathologic analysis (inflammatory cells and accumulation of fat) of liver tissues in each group was detected by H&E staining (magnification 5× and 10×). (a) NC (5×); (b) NC (10×); (e) NAFLD model group (5×); (f) NAFLD model group (10×); (i) MRP group (5×); (j) MRP group (10×). Effect of MRP on lipid accumulation was identified by oil red O (magnification 5× and 20×). (c) NC (5×); (d) NC (20×); (g) NAFLD model group (5×); (h) NAFLD model group (20×); (k) MRP group (5×); (l) MRP group (20×).

To visualize the effects of MRP supplementation on liver morphology in mice, liver tissues were stained with H&E and Oil Red O, respectively. As shown in Figure 2a,b, livers from mice on a normal diet showed a normal liver structure, with hepatocytes arranged uniformly in a cord‐like structure. As shown in Figure 2e,f, the hepatic cords of mice fed with HFHS were arranged irregularly and loosely, which suggested that the liver histology had been changed. After Oil Red O staining, mice fed with HFHS prominently presented with steatosis, accumulation of lipid droplet vacuoles, and increasing inflammatory cell infiltration compared to mice fed with a normal diet (Figure 2g,h). In contrast, macro‐ and micro‐lipid droplet vacuoles were almost decreased in the liver in these mice supplemented with MRP. Compared to the HFHS diet group, these mice exhibited a decreased abundance of informative cells, along with a more organized structure and morphology of liver cells (Figure 2i–l). These findings indicated that MRP supplementation alleviated the hepatic steatosis and liver damage induced by HFHS in mice.

### 3.3 Improvement of MRP on HFHS‐induced systematic inflammation, glucose level, and oxidation resistance in C57BL/6 mice

**FIGURE 3:**
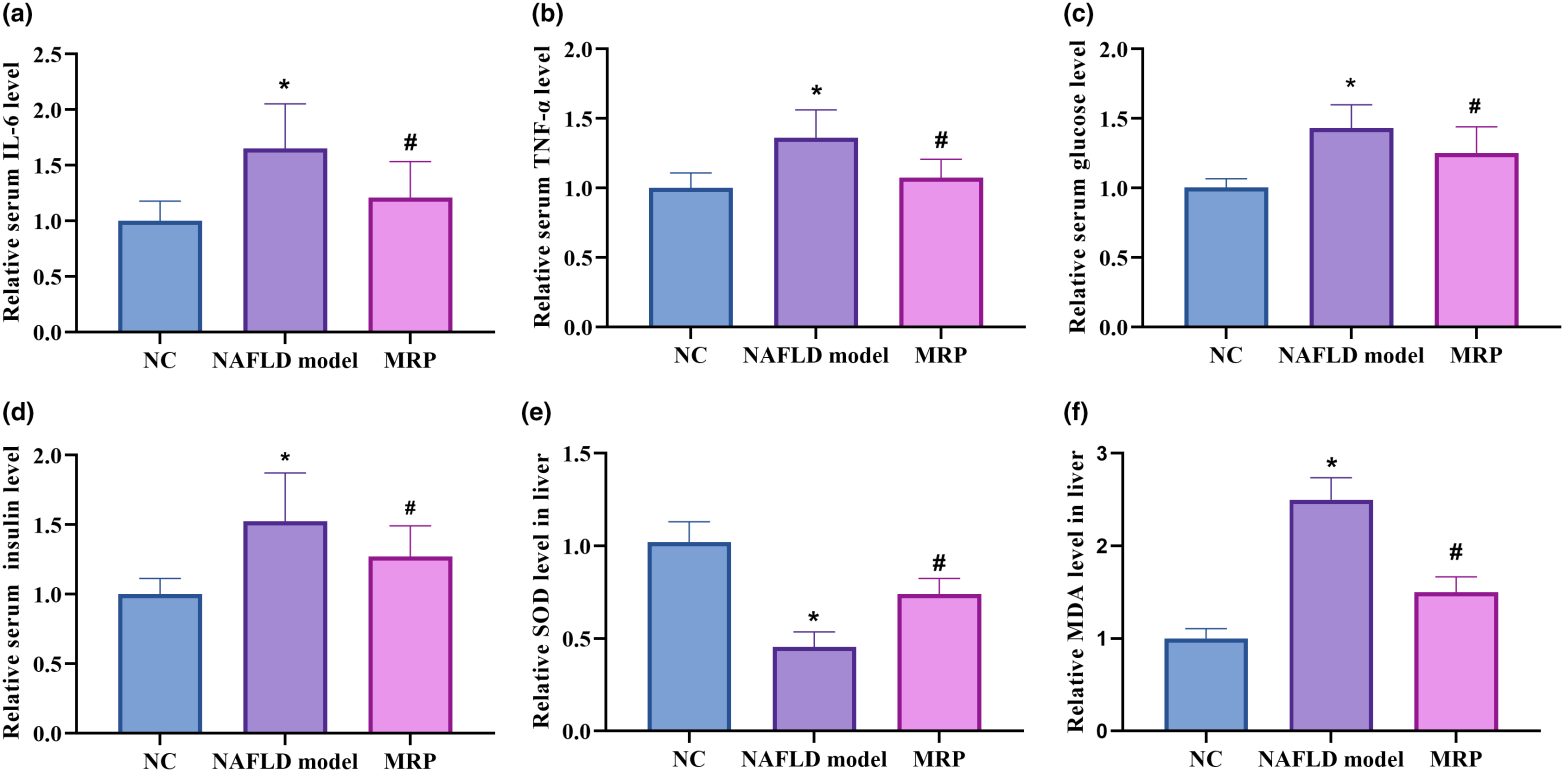
Effects of MRP supplementation on systemic inflammation, glucose tolerance, and liver antioxidant status. (a) The change in serum IL‐6 level. (b) The change in serum TNF‐α level. (c) The change in serum glucose level. (d) The change in serum insulin level. (e) The change in liver SOD level. (f) The change in liver MDA level.

The strength of the immune response is reflected by proinflammatory cytokine levels, which contribute to the advancement of NAFLD. For instance, an endotoxin, identified by toll‐like receptor 4 (TLR4), is a potent secondary hit that activates inflammasomes and causes progressive inflammatory damage. As depicted in Figure 3a,b, the livers of mice in the HFHS diet‐fed group significantly increased the concentration of IL‐6 and TNF‐α compared to mice fed a normal diet. Notably, it was remarkable that the supplementation of MRP effectively mitigated these elevations induced by the HFHS diet. Furthermore, mice fed the HFHS diet demonstrated significantly increased glucose intolerance and insulin levels compared to those fed a normal diet (Figure 3c,d). Conversely, enhanced glucose intolerance and insulin levels were reduced after MRP supplementation. Similarly, oxidation resistance in mice's livers was also improved by MRP (Figure 3e,f). These findings indicated that MRP supplementation reduced inflammation and improved glucose tolerance in mice fed HFHS diets.

### 3.4 Improvement of MRP on HFHS‐induced colon impairment in C57BL/6 mice

**FIGURE 4:**
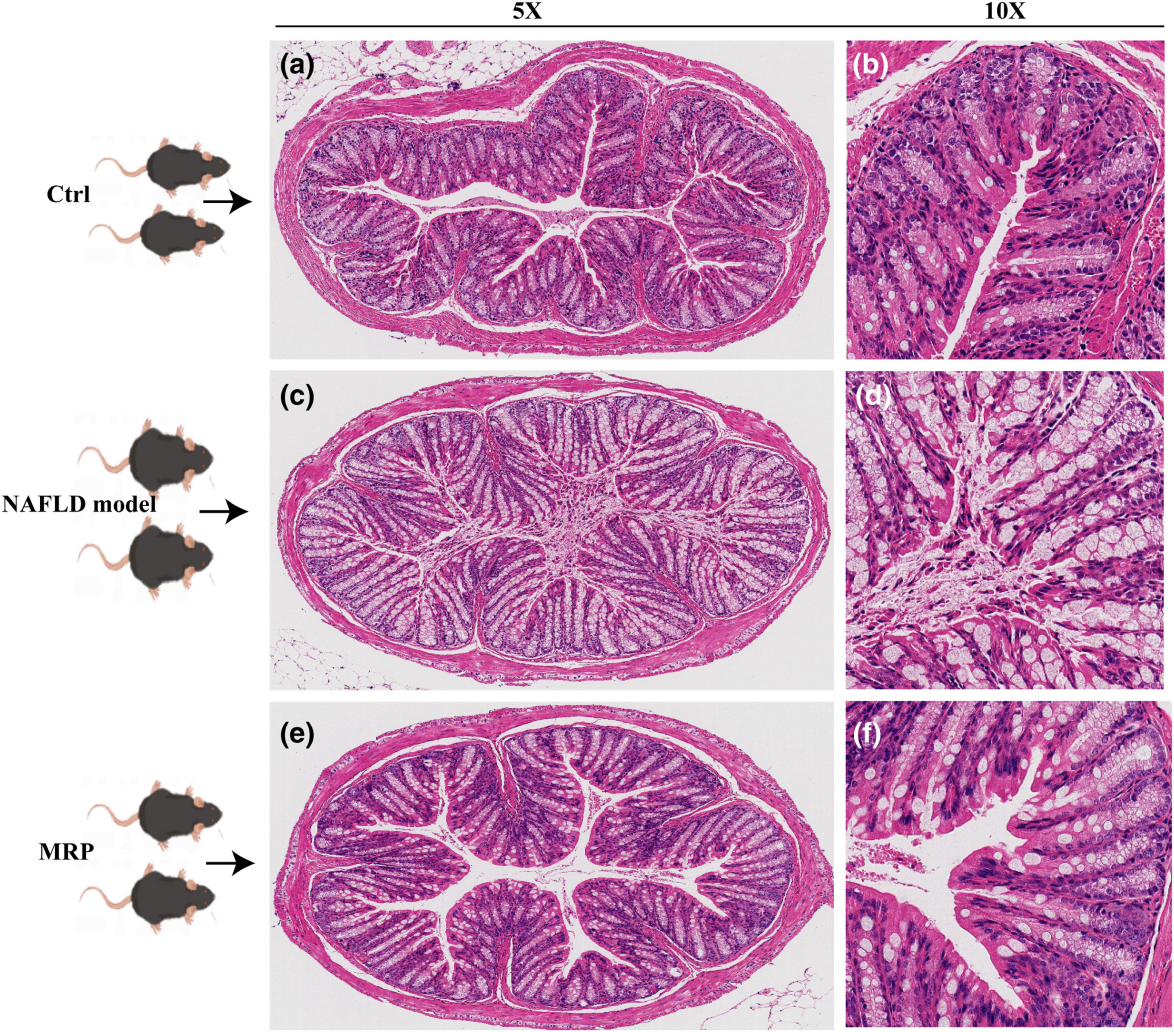
Effects of MRP supplementation on the colon in mice fed on a HFHS diet. Histopathologic analysis of colon tissues in each group was detected by H&E staining (magnification 5× and 10×). (a) NC (5×); (b) NC (10×); (c) NAFLD model group (5×); (d) NAFLD model group (10×); (e) MRP group (5×); (f) MRP group (10×).

Due to 90% of the microorganisms residing in the human body's colon, any changes in the composition and metabolites of the gut microbiota can impact the structure of the colon. This has been supported by evidence that NAFLD is associated with intestinal dysfunction and elevated permeability. Therefore, to further investigate the impact of MRP supplementation on the colon morphology of mice receiving a HFHS diet, the colon tissue morphology was assessed using H&E staining. As illustrated in Figure 4c,d, the HFHS diet profoundly impaired the colon's structure, including inflammation‐inducing cell infiltration, damaging mucosal epithelium and intestinal gland structure, crypt reduction, and mucosa absence. Notably, MRP supplementation effectively improved the severity of colon tissue damage, including alleviating colon shortening and reducing the infiltration of inflammatory cells in the colonic mucosa (Figure 4e,f). These results indicated that MRP supplementation improved the colon impairment induced by the HFHS diet.

### 3.5 MRP supplementation restored the gut microbiota in HFHS‐fed mice

**FIGURE 5:**
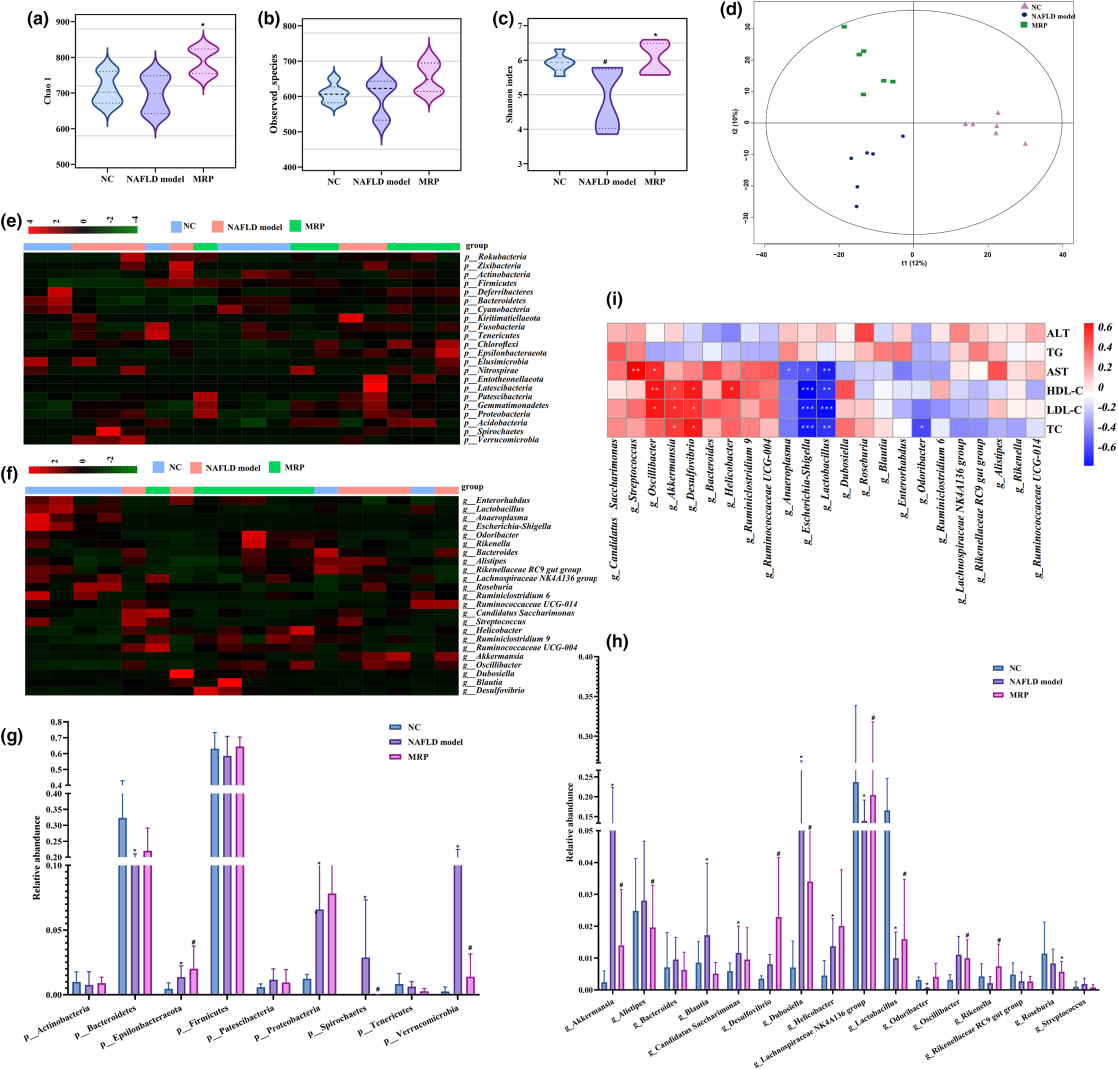
MRP supplementation restored HFHS‐induced gut microbiota dysbiosis. (a) The microbiota richness is measured by the chao1 index. (b) The microbiota richness is measured by the observed_species index. (c) The microbiota richness is measured by the Shannon index. (d) Principal component analysis (PCA) evaluated the richness of gut microbiota between NC, NAFLD model, and MRP supplementation in mice. (e) Heatmap plot of the bacteria at phylum level in fecal samples of the NC, NAFLD model, and MRP supplementation groups. (f) Heatmap plot of the bacteria at genus level in fecal samples of the NC, NAFLD model, and MRP supplementation groups. (g) The relative abundance of the bacteria in the phylum. (h) The relative abundance of the bacteria in the genus. (i) The correlation of bacteria in genus level and serum TG, TC, LDL‐C, HDL‐C, AST, and ALT. *p < .05, ^#^ *p* < .01. **p < .01, ***p < .001.

Due to the fact that gut microbial dysregulation is an important factor during the development of NAFLD, we explored the changes in the composition and abundance of the gut microbiota in mice fecal samples induced by a HFHS diet and MRP supplementation using 16S rRNA gene sequencing. As shown in Figure 5a–c, the Shannon index was noticeably reduced by the HFHS diet, while there was only a slight decrease in the Chao1 and Observed_species index. However, after MRP supplementation for 16 weeks, both the Chao1 and Shannon index showed a significant increase, while the Observed_species index exhibited an increasing trend without significance. As shown in Figure 5d, PCA analysis based on the abundance of OTUs revealed significant differences in the overall composition of the gut microbiota among the three groups. To examine the relative abundance of the gut microbiota, we performed heatmap analysis of OTUs at the phylum and genus levels. As shown in Figure 5e,g, the dominant phyla identified were *Firmicutes* and *Bacteroidetes*, both of which decreased in mice fed with a HFHS diet. However, after 16 weeks of MRP supplementation, the abundance of both *Bacteroidetes* and *Firmicutes* were increased. In addition, MRP supplementation led to a decrease in the abundance of *Patescibacteria*, *Spirochaetes*, and *Verrucomicrobia*, which were elevated by the HFHS diet. As shown in Figure 5f,h, at the genus level, the abundance of *Akkermansia*, *Blautia*, *Candidatus Saccharimonas*, *Dubosiella*, and *Oscillibacter* was significantly increased in mice fed with the HFHS diet compared to those fed with a normal diet. Conversely, MRP supplementation reduced the abundance of these elevated microbiota. Furthermore, MRP supplementation significantly increased the abundance of *Lactobacillus*, *Lachnospiraceae NK4A136 group*, and *Rikenella*, which were decreased by the HFHS diet.

To determine the association between the gut microbiota and liver function and lipid profiles, correlation analysis was performed at the genus level in the NC, NAFLD model group, and MRP groups. As shown in Figure 5i, *Streptococcus* and *Oscillibacter* were positively correlated with AST, while *Oscillibacter* and *Akkemansia* were also positively correlated with LDL‐C, HDL‐C, and TC. In contrast, *Lactobacillus* exhibited negative correlations with AST, LDL‐C, HDL‐C, and TC. These results further supported that gut microbiota imbalance contributed to liver injury and lipid profile disorders.

### 3.6 MRP supplementation improved the change of metabolites in serum and feces in HFHS‐fed mice

**FIGURE 6:**
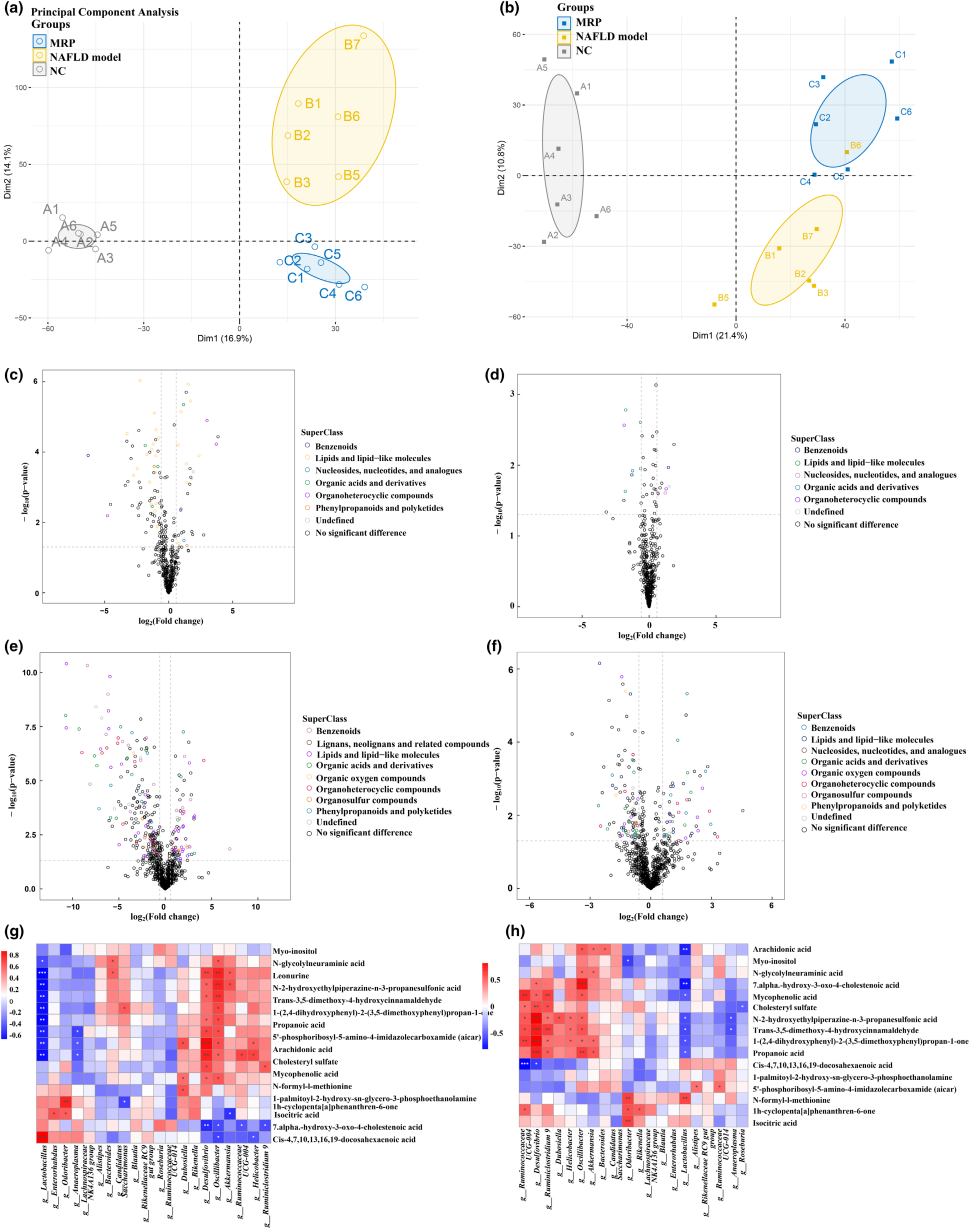
MRP supplementation balanced HFHS‐induced alteration of metabolic profiles in mice serum and feces. (a) Serum metabolites were significantly different between mice in the NC, NAFLD model, and MRP supplementation groups by principal component analysis. (b) Fecal metabolites were significantly different between mice in the NC, NAFLD model, and MRP supplementation groups by principal component analysis. (c) Volcano plot of serum metabolomics of mice in the NC and NAFLD model groups. (d) Volcano plot of serum metabolomics of mice in the MRP and NAFLD model groups. (e) Volcano plot of fecal metabolomics of mice in the NC and NAFLD model groups. (f) Volcano plot of fecal metabolomics of mice in the MRP and NAFLD model groups. (g) Correlation analysis of the association between the altered microbes and serum metabolites. (h) Correlation analysis of the association between the altered microbes and fecal metabolites. *p < .05, **p < .01, ***p < .001.

To study the metabolic disorder induced by the HFHS diet, the improvement of this disorder induced by MRP, we performed untargeted metabolomics analysis using serum and fecal samples from each group to measure the abundance of metabolites. The PCA score plot clearly demonstrated a distinct separation of serum and fecal metabolites between NC, NAFLD model, and MRP supplementation mice at the 16th week for MRP supplementation (Figure 6a,b). The volcano plot revealed that the different metabolites in fecal and serum samples induced by the HFHS diet were primarily lipids and lipid‐like molecules compared to the normal group (Figure 6c,e). Similarly, the predominantly altered metabolites induced by MRP supplementation were also lipids and lipid‐like molecules compared to the HFHS diet mice (Figure 6d,f).

Correlation analysis was also conducted to examine the potential associations between the gut microbiota altered by the HFHS diet and serum as well as fecal metabolites. As shown in Figure 6g, *Lactobacillus genera* were positively related to the level of *cis*‐4,7,10,13,16,19‐docosahexaenoic acid and negatively related to the level of *N*‐glycolylneuraminic acid, leonurine, *N*‐2‐hydroxyethylpiperazine‐n‐3‐propanesulfonic acid, trans‐3,5‐dimethoxy‐4‐hydroxycinnamaldehyde, 1‐(2,4‐dihydroxyphenyl)‐2‐(3,5‐dimethoxyphenyl) propan‐1‐one, propanoic acid, and arachidonic acid. In contrast, *Desulfovibrio* and *Oscillibacter* are positively correlated with these compounds and negatively related to *cis*‐4,7,10,13,16,19‐docosahexaenoic acid and 7‐alpha‐hydroxy‐3‐oxo‐4‐cholestenoic acid. Similarly, *Lactobacillus genera* was negatively related to the level of *N*‐2‐hydroxyethylpiperazine‐ n‐3‐propanesulfonic acid, trans‐3,5‐dimethoxy‐4‐hydroxycinnamaldehyde, 1‐(2,4‐dihydroxyphenyl)‐2‐(3,5‐dimethoxyphenyl)propan‐1‐one, propanoic acid, and arachidonic acid in fecal samples (Figure 6h).

**FIGURE 7:**
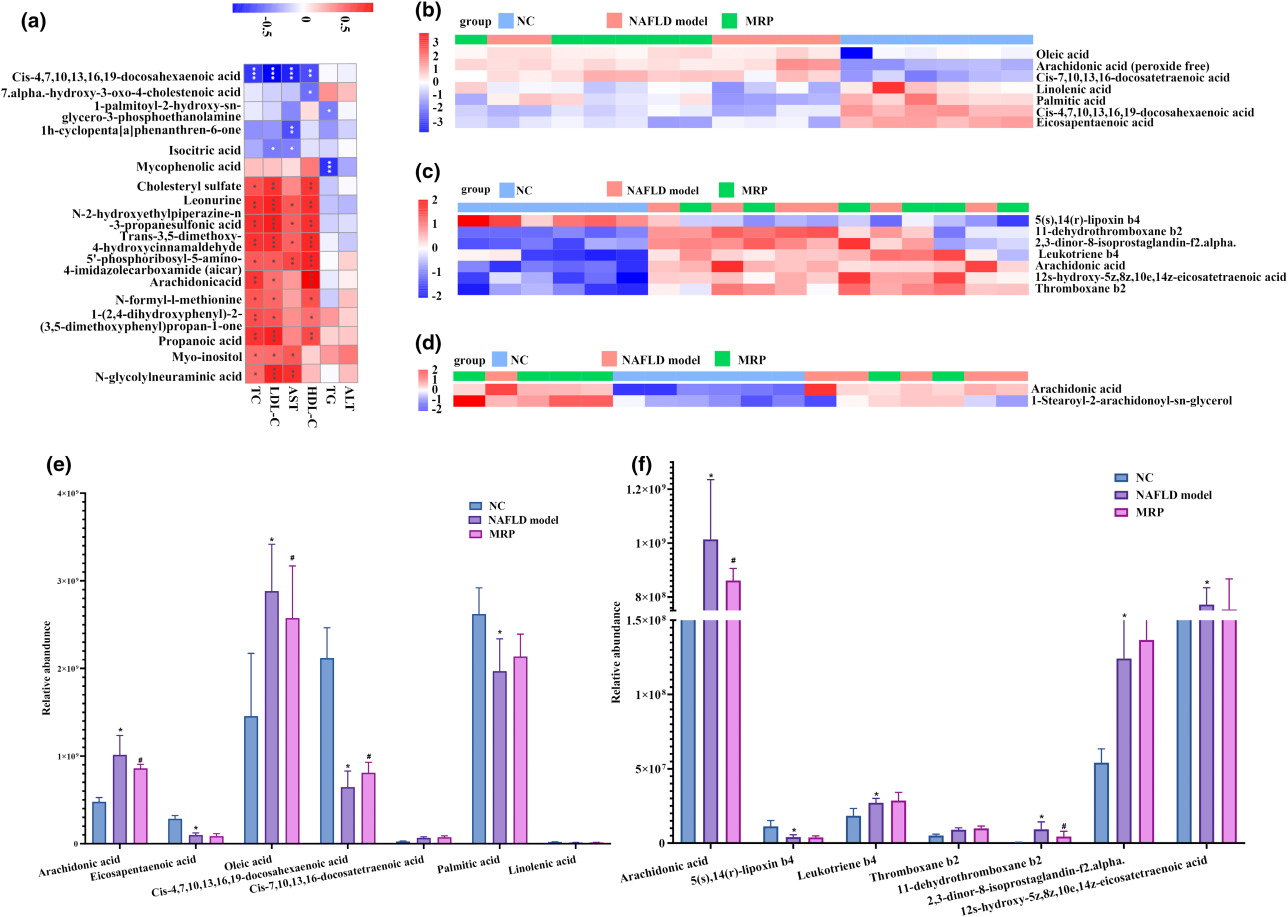
The change in top metabolites derived from the gut microbiota after MRP supplementation. (a) The correlation between significant differences in metabolites and serum TG, TC, LDL‐C, HDL‐C, AST, and ALT. (b) Pathway analysis of enriched metabolites in arachidonic acid metabolism. (c) Pathway analysis of enriched metabolites in the biosynthesis of unsaturated fatty acids. (d) Pathway analysis of enriched metabolites in regulation of lipolysis in adipocytes. (e) The relative abundance of metabolites in serum. (f) The relative abundance of metabolites in fecal. *p < .05, **p < .01, ***p < .001.

Furthermore, we explored the association between differentially expressed serum metabolites and liver function as well as lipid profiles. As shown in Figure 7a, *cis*‐4,7,10,13,16,19‐docosahexaenoic acid exhibited a negative correlation with AST, HDL‐C, LDL‐C, and TC, while isocitric acid showed a negative correlation with AST and LDL‐C. Lipid profiles were positively regulated by arachidonic acid, cholesteryl sulfate, and other metabolites. In addition, AST was positively regulated by leonurine, 5′‐phosphoribosyl‐ 5‐amino‐4‐imidazolecarboxamide, and *N*‐glycolyl neuraminic acid. These metabolites were then mapped to specific pathways using the KEGG database. Arachidonic acid, for example, is a key metabolite involved in arachidonic acid metabolism (map ID: mmu00590, Figure 7b). Moreover, arachidonic acid and *cis*‐4,7,10,13,16,19‐docosahexaenoic acid participate primarily in the biosynthesis of unsaturated fatty acids (map ID: mmu01040) (Figure 7c). Additionally, rachidonic acid and 1‐stearoyl‐2‐ arachidonoyl‐sn‐glycerol were involved in the regulation of lipolysis in adipocytes (map ID: mmu04923, Figure 7d). Arachidonic acid, increased by the HFHS diet and decreased by MRP supplementation, appeared to be a key metabolite during MRP ameliorating HFHS diet‐induced NAFLD (Figure 7e,f). These pathways shed light on potential molecular mechanisms underlying the attenuation of NAFLD progression through MRP supplementation.

## 4 DISCUSSION

In this study, we found that supplementation with MRP reduced the body weight, improved the lipid levels, and enhanced liver function induced by a HFHS diet. MRP supplementation also reduced the amount of lipid droplets, and ameliorated the disruption of hepatic portal architecture and colon morphology, inflammation, and glucose tolerance induced by the HFHS diet. In addition, MRP supplementation restored the dysbiosis of the gut microbiota caused by the HFHS diet. Specifically, it reversed the increased levels of *Akkermansia*, *Blautia*, *Candidatus saccharimonas*, *Dubosiella*, and *Oscillibacter* induced by the HFHS diet, while increasing the levels of *Lactobacillus*, *Lachnospiraceae NK4A136 group*, and *Rikenella*. This finding contrasts with previous studies based on mice fed a high fat diet (Wu et al., 2022; Yang et al., 2020; Zhou et al., 2019). Furthermore, MRP supplementation improved the serum and fecal metabolic profiles affected by the HFHS diet, including arachidonic acid, propanoic acid, and so on. The abundance of *Lactobacillus*, *Oscillibacter*, and *Akkemansia* was closely associated with liver function and lipid profiles. Consequently, fecal and serum metabolites, primarily involved in arachidonic acid metabolism, unsaturated fatty acid biosynthesis, and regulation of lipolysis in the adipocyte pathway, also exhibited strong associations with liver function and lipid profiles.

Recent studies have highlighted the prevalence of high‐fat diet (HFD) patterns in developing countries (Morris et al., 2015), leading to dyslipidemia and subsequent liver metabolic diseases, such as non‐alcoholic fatty liver disease (NAFLD) (Gao et al., 2020). The digestion and absorption of dietary fats primarily occur in the gut tract. Abnormal intake of fats can reduce the diversity of the gut microbiota and obviously increase the concentrations of LPS, which are known to be related to gut microbiota dysregulation (Lauterbach et al., 2020; Vatanen et al., 2016). Then, evaluated LPS levels can compromise the gastrointestinal tract, including destroying the intestinal barrier against invasion of harmful substances and nutrient absorption homeostasis in the small intestinal epithelium, thus contributing to the development of NAFLD (Zhang et al., 2021). This influence for NAFLD also arose from the interactive crosstalk between the liver and the complex microbial metabolites (Altay et al., 2019). For instance, the gut microbiota can either ameliorate or exacerbate liver diseases by influencing liver lipid metabolism, alcohol production, energy metabolism, intestinal permeability, and bile secretion (Guohong et al., 2019). In this study, we demonstrated that the altered diverse communities induced by a HFHS diet were associated with pathological change in NAFLD. Moreover, MRP supplementation could rebalance the gut microbiota and improve liver lipid metabolism. A previous study revealed that the depletion of *Akkermansia*, *Lactobacillus*, and *Bacteroides* with a high dietary cholesterol intake and the enrichment of *Helicobacter* with a high dietary fat and cholesterol intake were important in the role of gut microbiota in NAFLD‐HCC (X Zhang et al., 2021). In addition, *Desulfovibrio*, which was enriched in fecal samples of HFHC‐fed mice, exhibited a positive correlation with the high‐cholesterol diet as well as the level of cholesterol in serum and liver. Conversely, *Lactobacillus* showed a negative correlation with these factors, aligning with our findings. Previous research has also demonstrated a strong correlation between *Desulfo vibrionaceae* and obesity, metabolic syndrome, and inflammation (Loy et al., 2017; Ussar et al., 2015; Zhang et al., 2012).

*Desulfo vibrionaceae* and *Desulfo vibrio* have also been reported to be elevated in swine animals with NASH (Panasevich et al., 2018). Also, *Lactobacillus* has been shown to be protective against the NASH pathological process by positively influencing liver cholesterol metabolism (Naudin et al., 2020; Okubo et al., 2013). Consistent with our results, the first 8 weeks on a HFHS diet significantly reduced the relative abundance of *Lactobacillus*. A previous study reported that dietary supplementation with *Lactobacillus plantarum NA136* alleviated insulin resistance in a NAFLD model using C57BL/6J mice and increased the relative abundance of *Enterorhabdus* (Zhao et al., 2020). This supports our observation that MRP supplementation enhanced the abundance of *Lactobacillus*, which was decreased by the HFHS diet, thus improving NAFLD in mice. However, it was worth noting that *Lactobacilli* produced lactic acid by fermenting dietary ethanol, carbohydrates, and acetate, potentially contributing to liver injury and higher fibrosis scores in individuals with NASH (Duarte et al., 2018). Specifically, certain *Lactobacillus* species produced ethanol and converted it into acetaldehyde, leading to damage to the intestinal barrier, increased intestinal permeability, and the progression of NASH (Elshaghabee et al., 2016; Nosova et al., 2000; Rao, 2009). For instance, Val‐Val‐Tyr‐Pro (VVYP) may decrease the abundance of *Lactobacillus* to mitigate ethanol‐ or acetaldehyde‐induced intestinal permeability and endotoxemia, thereby improving HFD‐induced NAFLD (Xie et al., 2021).

Individuals with moderate steatosis exhibited a marked increase in the levels of *Firmicutes* (including *Streptococcus mitis* and *Roseburia inulinivorans*) and *Bacteroidetes* (such as *Barnesiella intestinihominis* and *Bacteroides uniformis*) compared to those without steatosis (Zeybel et al., 2022). In our study, we observed an elevation in the abundance of *Streptococcus*, *Alkaliphilus*, *Blautia*, and *Oscillibacter* due to the HFHS diet, which was subsequently reduced after MRP supplementation. It is worth mentioning that exercise has also been shown to effectively correct these microbial imbalances induced by the HFHS diet (Carbajo‐Pescador et al., 2019). However, in our study, the *Bacteroidetes* phylum was decreased by the HFHS diet and increased following MRP supplementation, which contrasted with a previous study based on *Monascus* pigment supplementation for high‐fat diet mice. Consequently, there are currently conflicting results regarding changes in the *Bacteroidetes* phylum in NAFLD‐related studies. For instance, one study showed that the abundance of *Bacteroides* is notably reduced in Chinese patients suffering from NAFLD compared with Western populations (Shen et al., 2017). However, other studies have shown that *Bacteroides* abundance was evaluated in NASH patients compared to those without NASH (Boursier et al., 2016). Additionally, the *Bacteroidetes* phylum was found to be higher in healthy individuals than in NAFLD patients (Shen et al., 2017). Furthermore, a slightly negative correlation was observed between higher fiber intake and the abundance of *Bacteroides uniformis*, while *Bacteroides* levels increased in individuals with obesity but decreased in lean participants following exercise intervention (Allen et al., 2018; Lin et al., 2018). These inconsistent findings may be due to variances in ethnicity, living environment, dietary habits, and lifestyle factors among the study subjects (Cheng et al., 2022).

Metabolites derived from gut bacteria are mainly produced in the gut tract through bacterial fermentation (Cummings et al., 1987). These metabolites are absorbed by colonocytes and then enter the bloodstream, eventually reaching the liver, where they can either benefit or contribute to the development of NAFLD (Ríos‐Covián et al., 2016). For instance, short‐chain fatty acids, amino acid catabolites, and bile acid, derived from the gut microbiota, can activate or inhibit their respective receptors, either relieving or exacerbating liver steatosis and inflammation (Ding et al., 2019). In this study, metabolomics profiling revealed that MRP supplementation led to changes in metabolites involved in arachidonic acid metabolism, biosynthesis of unsaturated fatty acids, and lipolysis in adipocytes. *Lactobacillus* genera was positively related to the level of *cis*‐4,7,10,13,16,19‐docosahexaenoic acid but negatively related to the level of N‐glycolylneuraminic acid, leonurine, 1‐(2,4‐dihydroxyphenyl)‐2‐(3,5‐dimethoxyphenyl) propan‐1‐one, and arachidonic acid. Conversely, *Desulfovibrio* and *Oscillibacter* positively correlated with these compounds and were negatively related to *cis*‐4,7,10, 13,16,19‐docosahexaenoic acid and 7‐alpha‐hydroxy‐3‐oxo‐4‐cholestenoic acid. Generally, arachidonic acid (AA), a subclass of polyunsaturated fatty acid (PUFA), is a pro‐inflammatory precursor that could potentially contribute to the development of NAFLD (Sztolsztener et al., 2020). Our results showed a positive relationship between AA and lipid profiles. Another study suggested that a higher level of AA was associated with an evaluated level of ALT, with each standard deviation increase in AA level corresponding to a 0.21 U/L change in ALT level (Chen et al., 2023). The synthesis of PUFA in the liver involves a microsomal process dependent on acyl‐CoA desaturases and chain elongation reactions. During this process, l‐palmitoyl‐sn‐glycero‐3‐phosphocholine consistently inhibited the *p*‐oxidation of linoleic acid and arachidonic acid due to substrate esterification by microsomal l‐acylsn‐glycero‐3‐phosphocholine acyltransferase. Furthermore, the *p*‐oxidation cycle of 7,10,13,16‐docosatetraenoic acid was also independent of l‐acyl‐sn‐glycero‐3‐phosphocholine and microsomes (Baykousheva et al., 1994). Restoring the abundance of gut microbiota‐derived metabolites might be one of the mechanisms by which MRP exerted its protective effect against NAFLD. Consistently, MRP supplementation influenced the levels of metabolites involved in arachidonic acid metabolism and the biosynthesis of unsaturated fatty acid pathways, potentially by upregulating the expression of SOD‐2 genes and downregulating GPX genes in liver tissue (Chaudhary et al., 2022).

However, this experiment had certain limitations that needed to be addressed. First, the gut microbiota composition is species‐diverse and undergoes dynamic and daily rhythmic changes (Gerber et al., 2012). Therefore, it is essential to analyze the variations in gut microbiota across different time periods and species. Second, previous studies have shown that a HFHS diet combined with MRP supplementation enriched the abundance of *Ethanologenbacterium harbinense*, *Oscillibacter* sp., *Coprococcus catus*, and *Clostridium leptum*, which was negatively related to the liver index. Additionally, *Clostridium saccharolyticum* and *Acetanaerobacterium elongatum* are positively associated with TG, TC, and bile acid levels (Zhou et al., 2019). However, in our study, we did not observe the enrichment and correlation of these microbial species, except for *Oscillibacter* sp., after MRP supplementation in mice fed the HFHS diet. Third, our study only provided a preliminary evaluation of the potential mechanisms underlying the beneficial effects of MRP supplementation on HFD‐induced NAFLD through gut microbiota and metabolism. Further research is necessary to investigate the specific relationship between MRP supplementation, gut microbiota, and metabolites.

## 5 CONCLUSION

This study conducted a comprehensive multi‐omics analysis to investigate the beneficial effects of MRP supplementation on improving NAFLD induced by a HFHS diet in a mouse model. Through our research, we identified key gut microbiota and metabolite features altered by MRP supplementation, which played a crucial role in ameliorating NAFLD. Our findings confirmed changes in microbial composition and metabolic disruptions associated with NAFLD triggered by the HFHS diet. Additionally, we observed that MRP could modulate arachidonic acid metabolism, unsaturated fatty acid biosynthesis, and adipocyte lipolysis, thus mitigating the progression of NAFLD. These results offered mechanistic insights into the positive impact of MRP on NAFLD patients based on microbiota composition, underscoring the potential of MRP as a functional food in the future.

## AUTHOR CONTRIBUTIONS

**Wenyan Gao:** Conceptualization (equal); supervision (equal); writing – review and editing (equal). **Xinghao Chen:** Data curation (equal); formal analysis (equal). **Shaokang Wu:** Investigation (equal). **Lu Jin:** Software (equal); validation (equal). **Xu Chen:** Investigation (equal); methodology (equal). **Genxiang Mao:** Supervision (equal). **Xiaoqing Wan:** Writing – review and editing (equal). **Wenmin Xing:** Writing – original draft (equal).

## FUNDING INFORMATION

This study was supported by Projects of Hangzhou Health Science and Technology Plan (ZD2022005), Hangzhou Administration of Traditional Chinese Medicine (A20220018), Hangzhou Medical College (KYYB202207), Zhejiang Provincial Administration of Traditional Chinese Medicine (GZY‐ZJ‐KJ‐24055), National Key Research on Modernization of Traditional Chinese Medicine (2022YFC3501802), the Program of the Health Bureau of Zhejiang Province (2021ZB009, 2021ZB079, 2021KY635), and the Zhejiang University of Medicine Aging Disease Prevention Research Center (2022060003).

## CONFLICT OF INTEREST STATEMENT

The authors declare no conflict of interest.

## ETHICS STATEMENT

All animal experiments were approved by the Animal Experimental Ethics Committee of Hangzhou Medical College.

## Data Availability

The original data are available by email to the corresponding author.
